# *In vitro* immunomodulatory potential of *Artemisia indica* Willd. in chicken lymphocytes

**DOI:** 10.14202/vetworld.2018.80-87

**Published:** 2018-01-28

**Authors:** Pushpa Ruwali, Tanuj Kumar Ambwani, Pankaj Gautam

**Affiliations:** 1 Department of Biotechnology, Graphic Era University, Dehradun - 248 002, Uttarakhand, India; 2 Department of Veterinary Physiology and Biochemistry, College of Veterinary & Animal Sciences, G. B. Pant University of Agriculture & Technology, Pantnagar - 263 145; Uttarakhand, India

**Keywords:** *Artemisia indica* Willd, immunomodulation, *in vitro* chicken lymphocytes, lymphocyte proliferation assay, plant extract

## Abstract

**Aim:**

Evaluation of the in vitro immunomodulatory potential of Artemisia indica Willd. methanolic extract in chicken lymphocyte culture system through lymphocyte (B and T cells) proliferation assay, after standardizing the maximum non-cytotoxic dose (MNCD) in chicken lymphocytes.

**Materials and Methods:**

Fresh aerial parts of A. indica Willd. (family: Asteraceae) specimens were collected (altitude 1560 m), gotten authenticated, processed, dried, and Soxhlet extracted to yield methanolic extract (AME). Chicken splenocytes were isolated from spleens collected from healthy birds; lymphocytes were separated by density gradient centrifugation, percentage cell viability determined and final cell count adjusted to 10^7^ cells/ml in RPMI-1640 medium. MNCD of AME in chicken lymphocytes was determined through 3-(4,5-dimethylthiazol-2-y1)-2,5-diphenyltetrazolium bromide dye reduction assay. Immunomodulatory potential of AME was evaluated through lymphocytes proliferation or B and T cells blastogenesis assay in the presence of appropriate mitogens, namely, lipopolysaccharide (LPS) and concanavalin A (Con A), respectively.

**Results:**

Maximum concentration of AME exhibiting 100% cell viability (MNCD) was 200 μg/ml and was selected for further in vitro analysis. The in vitro exposure of chicken lymphocytes to 200 µg/ml dose of AME, resulted in significant (p<0.05) upregulation of 11.76% in B cell proliferation in the presence of B cell mitogen (LPS) and a significant (p<0.05) increase of 12.018% T cells proliferation in the presence of the mitogen (Con A), as compared to the control.

**Conclusion:**

The significant upregulation in the proliferation of two major cell types modulating the immune system is an indication of the immunostimulatory potential of the plant. It would be worthwhile to further evaluate A. indica on relevant immunomodulatory aspects, especially the in vivo studies in a poultry system.

## Introduction

The immune system, more than any other system in the body, is central to one’s health and well-being because it affects every other part of the body. Immune system is not only crucial to homeostasis; it plays a key role in the defense against infections and cancers. Its integrity and efficiency are important during chemotherapeutic intervention for the treatment of many diseases [1]. Immunomodulation is a procedure that can alter the immune system of an organism by interfering with its function. If it results in an enhancement/decrease of immune reaction, it is named as “Immunomodulatory” [2].

Plants have played a significant role in maintaining human and animal health and improving the quality of life for thousands of years. Modulation of immune responses to alleviate disease has been one mechanism of interest. A number of medicinal plants have been shown to stimulate or inhibit the immune system [3].

A medicinal plant genus *Artemisia* (Asteraceae), popularly known as “Sage Brush” or “Wormwood,” has been used extensively in folk medicine and as food by many cultures since times immemorial [4]. A good amount of literature is available proclaiming the ethnoveterinary usage of *Artemisia* species throughout world including India, China, Japan, Pakistan, several middle-east, and African countries, in livestock including poultry as anthelmintic, antiprotozoal for blood parasite (both intra- and extra-cellular), anticoccidial, acaricidal, antispasmodic, diuretic, and cholagogue, against veterinary tumors and sarcomas and as fodder for ruminants [5].

*Artemisia* is a diverse and economically important genus, and it has more than 500 species reported in the world and out of which about 47 species, are found in India [6,7]. In India, *Artemisia indica* Willd. (hereinafter *A. indica*) vernacularly known as “*titépâti*,” is a perennial herb found in the western Himalayas and is one of the most utilized locally as a traditional medicinal plant and animal feed, especially in the *Kumäuñ* hills (Uttarakhand, India), though, surprisingly, is also one of the lesser studied one [4]. A fair number of reports confirm the use of *A. indica* as a culinary herb and a food plant all over the world, including India, Pakistan, Nepal, and Japan. Ethnomedicinally, *A. indica* has been employed by local people to alleviate chronic fever, dyspepsia, hepatobiliary ailments, as well as an anthelmintic, antiseptic, antispasmodic, emmenagogue, expectorant, and stomachic [4,8]. Members of the genus *Artemisia* have been known to possess phytochemicals responsible for its immunomodulatory potential [3]. Numerous species of *Artemisia* have been reported to exhibit anti-inflammatory activity [9-11].

According to the WHO, across the world, traditional medicine is either the mainstay of health care delivery or serves as a complement to it. Traditional medicines, of proven quality, safety, and efficacy, contribute to the goal of ensuring that all people have access to care. For many millions of people, herbal medicines, traditional treatments, and traditional practitioners are the main source of health care, and sometimes the only source of care. This is care that is close to homes, accessible, and affordable. It is also culturally acceptable and trusted by large numbers of people. The affordability of most traditional medicines makes them all the more attractive at a time of soaring healthcare costs and nearly universal austerity. Traditional medicine also stands out as a way of coping with the relentless rise of chronic non-communicable diseases [12]. Hence, the need for the search for newer herbal sources and more importantly the validation of their ethnomedicinal claims on the modern scientific platform.

Meager information is available on the immunomodulatory activity of *A. indica*. In spite of sincere efforts, authors could not find any reports on the *in vitro* immunological studies of *A. indica* extracts in avian system, and to the best of the knowledge, the present communication is the first report of the *in vitro* immunomodulatory potential of *A. indica* Willd. extract in chicken lymphocytes.

## Materials and Methods

### Ethical approval

This research was carried out after procuring the necessary approval from the Institutional Animal Ethics Committee, College of Veterinary and Animal Sciences, Govind Ballabh Pant University of Agriculture and Technology, Pantnagar, Uttarakhand, India.

### Plant material and preparation of plant extract

Fresh aerial parts of *A. indica* Willd. (family: Asteraceae) specimens were collected at an altitude of 1560 m, strictly abiding by the standard precautions in the month of June from the *Kumäuñ* hills of Okhalkanda block (latitude 29°39’ N and longitude 79°67’ E), near Bhimtal, district Nainital, Uttarakhand (India). The plant specimens were authenticated in the Botanical Survey of India (BSI), Northern Circle, Dehradun (Uttarakhand). A voucher specimen (Acc. no. 114879) was deposited at the herbarium of BSI. The extraction of dried and finely powdered plant material was done by Soxhlet extraction (1:10 w/v extract: solvent) with methanol to yield *A. indica* methanol extract (AME). Extract preparation in detail is described elsewhere [4].

### Chicken lymphocytes isolation and cell viability assay

Chicken splenocytes were isolated from spleens collected from healthy birds, under laminar air flow as per standard procedure [13]. Lymphocytes were separated by density gradient centrifugation (Hisep-LSM, HiMedia, India) as per the method described by Rose and Friedman [14]. Percentage cell viability was determined by 0.1% Trypan blue dye exclusion test using hemocytometer [15], and final cell count was adjusted to 10^7^ cells/ml in RPMI-1640 medium with antibiotic and antimycotic solution supplemented with 10% Fetal Bovine Serum (all HiMedia, India).

### Determination of non-cytotoxic dose of AME in chicken lymphocyte cell culture system

The isolated lymphocytes were exposed to various dilutions of AME (prepared in RPMI- 1640) in triplicate in 96 well tissue culture plate. Maximum non-cytotoxic dose (MNCD) of AME in chicken lymphocytes was determined through 3-(4,5-dimethylthiazol-2-y1)-2,5- diphenyltetrazolium bromide (MTT) (Sigma) dye reduction assay [16]. The extract (AME) was used in concentrations ranging from 0.1 μg/ml to 1.6 mg/ml (namely, 0.1 μg/ml, 0.5 μg/ml, 1 μg/ml, 5 μg/ml, 10 μg/ml, 50 μg/ml, 100 μg/ml, 200 μg/ml, 300 μg/ml, 400 μg/ml, 500 μg/ml, 600 μg/ml, 700 μg/ml, 800 μg/ml, 900 μg/ml, 1 mg/ml, 1.2 mg/ml, 1.4 mg/ml, and 1.6 mg/ml). The maximum concentration of AME showing 100% cell viability (MNCD) in comparison to control was selected for further experimentations.

### Determination of AME induced immunomodulation through lymphocytes proliferation assay

Immunomodulatory potential of AME was evaluated through lymphocytes proliferation or B and T cells blastogenesis assay in the presence of appropriate mitogens, namely, lipopolysaccharide (LPS, Sigma) and concanavalin A (Con A, HiMedia). Lymphocyte proliferation assay (LPA) was carried out as per the method described by Lee *et al*. [17]. Briefly, the cells were cultured in wells (in triplicate) using 100 µl of cell suspension in the presence of either LPS or Con A in respective wells of 96 well flat bottom sterile microtiter plate in the presence and absence of AME. The plate was sealed with protective cellophane tape and was incubated for 68 h at 37°C in 5% CO_2_ gas tension in the incubator. At the end of the incubation, 50 µl of MTT (5 mg/ml) dye was added to all the wells under a sterile condition in the laminar air flow and further incubated for 4 h at 37°C in 5% CO_2_ gas tension. Contents of the plate were removed slowly, and 100 µl of dimethyl sulfoxide was added to all the wells and mixed by gentle pipetting.

The absorbance of each well was measured at 570 nm wavelength using Microplate ELISA Reader. Mean optical density (O.D.) was calculated by subtracting mean O.D. of control wells from the mean O.D. of mitogen-stimulated culture wells, as per the following formula.

Mean optical density (O.D.)=Mean O.D. of mitogen-stimulated culture well−Mean O.D. of control

### Statistical analysis

All experiments were performed in triplicate and data were reported as mean±standard deviation. The relevant data were statistically analyzed by CRD (completely randomized block design) using ANOVA [18]. The critical difference at 5% (p<0.05) level of significance for each character was worked out for comparing the significance of the treatment means. The differences between the control group and treatment groups were determined using t-test and p<0.05 was regarded as significantly different.

## Results

The AME was assessed for it’s *in vitro* immunomodulatory potential in chicken lymphocytes culture, and for any such *in vitro* cell culture studies, it becomes imperative to standardize the “non-cytotoxic dose” in the selected system.

### Non-cytotoxic dose of AME in chicken lymphocytes culture

**Figure-1:**
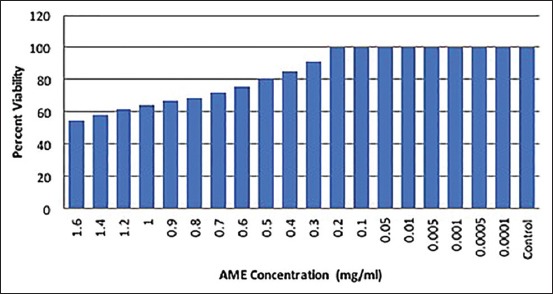
Non-cytotoxic dose of methanolic extract of *Artemisia indica* in chicken lymphocytes culture by 3-(4,5-dimethylthiazol-2-y1)-2,5-diphenyltetrazolium bromide cytotoxicity assay.

The chicken lymphocytes were exposed to various dilutions of AME to determine its MNCD for ensuing *in vitro* studies. The results obtained are presented in Table-1 and Figure-1. The data indicated dose-dependent cytotoxicity induced by AME in chicken lymphocytes culture, namely, higher the concentrations of AME, i.e., ranging from 0.3 mg/ml to 1.6 mg/ml, more was the cytotoxicity. AME displayed maximum cytotoxicity at the highest concentration of 1.6 mg/ml used in the study with 54.72% cytotoxicity. AME concentrations ranging from 0.2 mg/ml (200 μg/ml) to 0.0001 mg/ml (0.01 μg/ml) showed 100% cell viability. Since the maximum concentration of AME that showed 100% cell viability was 0.2 mg/ml (200 μg/ml), thus it was selected for further *in vitro* analysis. This MNCD (200 μg/ml) for AME was then used for evaluation of its immunomodulatory potentials through B and T cells proliferation in the presence of respective mitogens, namely, LPS (B cells) and Con A (T cells).

**Table 1:** The maximum non-cytotoxic dose of AME in chicken lymphocytes culture system.

**AME concentration (mg/ml)**	**1.6**	**1.4**	**1.2**	**1.0**	**0.9**	**0.8**	**0.7**	**0.6**	**0.5**	**0.4**

Mean OD (570 nm)± SD*	0.162±0.075	0.173±0.011	0.183±0.005	0.190±0.007	0.197±0.002	0.203±0.002	0.213±0.006	0.223±0.006	0.239±0.003	0.252±0.010
% viability rate^#^	54.72	57.66	61.82	64.18	66.55	68.58	71.95	75.33	80.74	85.13

**AME concentration (mg/ml)**	**0.3**	**0.2**	**0.1**	**0.05**	**0.01**	**0.005**	**0.001**	**0.0005**	**0.0001**	

Mean OD (570 nm)± SD*	0.269±0.008	0.296±0.002	0.297±0.004	0.298±0.004	0.300±0.007	0.302±0.006	0.305±0.006	0.308±0.005	0.311±0.003	
% viability rate^#^	90.87	100	100	100	100	100	100	100	100	

Cd at 1%=0.014; Cd at 5%=0.010; SEM=0.003. *p<0.05; #Percent viability rate calculated as {OD (treated)/OD (Control)} 100%. AME=*Artemisia indica* methanol extract, SD=Standard deviation, SEM=Standard error of the mean

### Effect of AME on B cells proliferation

**Figure-2:**
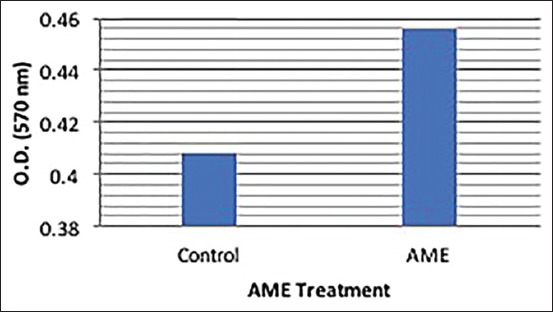
Effect of methanolic extract of *Artemisia indica* on B cell proliferation in the presence of the mitogen (lymphocyte proliferation assay).

The results of *in vitro* effects of AME on B cell proliferation in chicken lymphocytes are depicted in Table-2 and Figure-2. The *in vitro* exposure of chicken lymphocytes to 200 µg/ml dose of AME, showed significant (p<0.05) increase of 11.76% in B cell ­proliferation in the presence of B cell mitogen (LPS).

**Table 2:** Effects of AME on B cell proliferation.

Control/treatment	Mean OD (570 nm)±SD	% proliferation	% change in proliferative index
Control	0.408±0.006*	100	0
Treatment with AME	0.456±0.007*	111.76	+11.76
t value	10.78253	*	

* p<0.05. AME=*Artemisia indica* methanol extract, SD=Standard deviation

### Effect of AME on T cells proliferation

**Figure-3:**
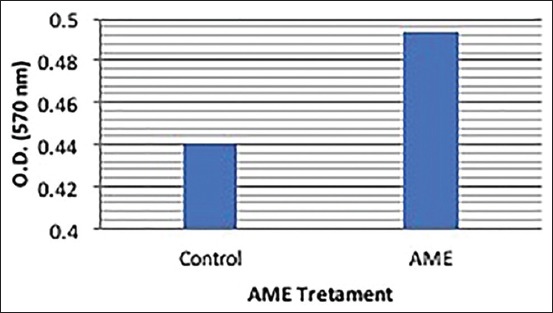
Effect of methanolic extract of Artemisia indica on T cell proliferation in the presence of the mitogen (concanavalin A).

AME treated cells displayed a significant (p<0.05) increase of 12.018% T cells proliferation as compared to the control in the presence of the mitogen (Con A) (Table-3 and Figure-3).

**Table 3:** Effects of AME on T cell proliferation (Con A).

Control/treatment	Mean OD (570 nm)±SD	% proliferation	% change in proliferative index
Control	0.441±0.007*	100	0
Treatment with AME	0.494±0.007*	112.018	+12.018
t value	18.00912	*	

* p<0.05. AME=*Artemisia indica* methanol extract, SD=Standard deviation

## Discussion

Poultry is an important source of food and nutrition not only in ethnic and tribal societies since ancient history but also in the developed world today. Birds have played a central role in many biological disciplines, particularly ecology, evolution, and behavior. The chicken, as a model vertebrate, also represents an important experimental system for developmental biologists, immunologists, cell biologists, and geneticists [19]. Besides this, Indian Poultry Industry is 5000 years old and has been providing nutritional security to the poor and also offering employment to millions of people in rural and urban areas [20]. All this formed the motivational ground to choose chicken as the animal model system for evaluating the immunomodulatory potential of *A. indica*.

Immunomodulation is one of the main targets for synthetic drugs and chemicals. However, its high cost, anticipated toxicity, and adverse effects render it undesirable for patients. In contrast, the use of herbal plants as health promoters is gaining increasing attention in both consumers and scientific circles [21]. Plants produce a great variety of organic compounds that are not directly involved in primary metabolic processes of growth and development. The roles these natural products or secondary metabolites play in plants have only recently come to be appreciated in an analytical context of modern science [22]. In recent years, the medicinal values of phytochemicals have aroused much concern, especially as immunomodulators or agents used for the adjunctive treatment of cancer [23].

Members of the genus *Artemisia* have been reported to possess phytochemicals responsible for its immunomodulatory potential, with the majority of reports describing its anti-inflammatory properties [3]. *Artemisia annua* extracts contain Artemisinin, has the formula C_15_H_22_O_15_ and contains a peroxide bridge (C-O-O-C), have anti-inflammatory properties [11,24]. Methanol extracts of eight different *Artemisia* species showed anti-inflammatory activity *in vitro* [9]. The anti-inflammatory activity of the methanolic leaf extract of *Artemisia vulgaris* has been established using cotton pellet granuloma method [10]. Methanolic extract of aerial parts of *A. vulgaris* has potent analgesic and anti-inflammatory activities [25].

Even though “*titépâti*” (*A. indica*) has been used as an anti-inflammatory and adaptogen by the local people in *Kumäuñ* hills for human and animal use since ages, meager scientific literature is available on the immunomodulatory aspect of *A. indica*. The exhaustive literature search yielded only an isolated report of *A. indica* in which methanolic extract of *A. indica* was evaluated for anti-inflammatory action by carrageenin-induced rat paw edema and was reported to be anti-inflammatory and analgesic properties in a dose-dependent manner [26].

Cellular proliferation is an essential feature of the adaptive immune response. The lymphocyte proliferation is an important index to evaluate cellular immunity, and the lymphocyte proliferation rate can directly reflect the strength of cellular immunity [27]**.** As mentioned above, the LPA in the presence of AME and respective mitogens resulted in a significant (p<0.05) upregulation of both B cells (11.76%) and T cells (12.018%) populations. Although no similar/related report could be found regarding *A. indica* in the literature, there are relevant reports on few other species of *Artemisia*. The effects of methanolic extract and leaf powder of *A. annua* were studied on performance, cellular and humoral immunity in 240 Cobb broiler chicks in a completely randomized design with the conclusion that the extract and leaf powder increases performance, cellular and humoral immunity of broilers [28]. Polysaccharide fractions purified from *Artemisia selengensis* and *Artemisia iwayomogi* exhibited immunomodulatory and antitumor properties [29] and extended the survival of murine spleen cells *in vitro* [30]. The immunomodulatory effects assessed by splenocyte proliferation and the cytokine secretion suggested the polysaccharides fractions from Moxa (*Artemisia argyi*) leaf are good immune enhancers [31].

Immunomodulatory activity of plants has been attributed eventually to the presence of various phytochemicals [32,33]. In general, there are at least two basic approaches which may be responsible to such activity, namely, these phytochemicals act directly on the mediators responsible for immune system or indirectly by the virtue of their antioxidative property. Probably, the actual mechanism of the immunomodulatory activity by these two basic approaches is mutually supportive and quite possibly synergistic as well [32]. Various plants derived compounds, namely, triterpenoids [34], flavonoids [35], saponins [36], or triterpenoid saponins [37] are reported to exhibit anti-inflammatory property. In one of our earlier reports pertaining to the qualitative and quantitative phytochemical evaluation of various extracts of *A. indica*, the AME revealed to possess various phytochemicals including major secondary metabolites, namely, carbohydrates, reducing sugars, amino acids, saponins, flavonoids, alkaloids, tannins, sterols, triterpenoids, phenolics, and glycosides [4].

Literature supports and suggests that there is a strong correlation between antioxidative properties of a plant and its immunomodulatory, particularly it’s immunostimulatory potential [38-40]. It is now an established fact, that natural antioxidants from plants such as polyphenols play an important role in the protection of cells from oxidative damage and consequently induce anticancer activities including proapoptotic, DNA damaging antiangiogenic, and immunostimulatory effects [41].

Many studies suggest that endogenous antioxidants, or exogenous antioxidants present in the diet, can function as free radical scavengers and improve human and animal health [42,43]. There is an adequate amount of data indicating that the functions of the human immune system depend on the intake of micronutrients, which can act as antioxidants. Several clinical trials have found that antioxidant supplementation can significantly improve certain immune responses [44]. There are numerous reports which confirm the positive effects of natural flavonoids on immune system of different species, including chicken [28,45,46]. We have earlier demonstrated that AME is rich in phenolics with a total phenolic content of 255.5±6.71 mg of GAE/g and a total flavonoid content of 161.2±4.95 mg of QE/g [4]. We further evaluated the *in vitro* antioxidative potential of various extracts of *A. indica* through DPPH, H_2_O_2_, NO scavenging and total antioxidant capacity assays and here also AME exhibited significantly better antioxidant activity as compared to other extracts [47].

It has been pointed out that the improvement of innate resistance to infectious disease is one of the mechanisms for the achievement of better health status and higher productivity in poultry. The immune system could be activated not only through infectious agents but also through injection of LPSs isolated by bacteria [48,49]. The immunomodulatory role of plant polysaccharides is quite well described [50,51], including the anti-inflammatory effect [52]. *Artemisia* species used in traditional medicine have shown to contain polysaccharides with a wide variety of biological properties [53]. A water-soluble carbohydrate fraction isolated from *A. iwayomogi* has been reported to modulate the functional differentiation of bone marrow-derived dendritic cells [54] and showed immunomodulating and antitumor activities in mice [29]. Antitumor and immunomodulatory activities of a polysaccharide (FAAP-02) isolated from *A. argyi* were evaluated, and the results indicated that the antitumor activity of FAAP-02 might be associated with its immunostimulatory effects [55]. Polysaccharide fractions from *A. tripartita* exhibited potent phagocyte immunomodulatory activity, reactive oxygen species scavenging and complement-fixing activity [56]. The immunostimulating effects exhibited by AME through LPA could also be attributed to the polysaccharides either alone or in conjunction with various phenolics.

Here it is quite appropriate to mention an important consideration. It has been pointed out in the litera­ture that according to diverse ethnomedicinal practices world over, especially the holistic approach of Ayurveda, the interplay of the various bioactive components and diverse biomolecules in a plant is of paramount importance in its medicinal and pharmaceutical activity [32]. The crude extracts of plants are pharmacologically more active than their isolated active principles probably due to the synergistic effects of various components present in the whole extract [57]. Another worthwhile consideration would be that according to epidemiological studies and trials on humans, it is evident that the health benefits of phytochemicals were observed predominantly when being consumed within their natural food matrices (fruits, vegetables, grain, etc.) [58].

Nevertheless, looking to the potential immunostimulatory activity of AME in chicken lymphocytes, it would be worthwhile to further evaluate/study the needed aspects such as the biomolecular fractionation studies and also bioavailability (absorption, metabolism and cellular, and tissue distribution) in a suitable system, establishing whether the *in vitro* effects are applicable to the situation *in vivo*. This would further pave the way to use/develop *A. indica* as a natural source of an immunomodulator and adaptogen in the form of a feed additive or food supplement for animals and humans.

## Conclusion

Literature and ethnomedicinal experiences and beliefs support and suggest that members of genus *Artemisia* are excellent candidates to be developed into herbal immunomodulating source species/agent. Methanolic extract (AME) of *A. indica*, a plant used as a traditional medicine and animal feed in the *Kumäuñ* hills (Uttarakhand, India), was evaluated for its immunomodulatory potential employing standard LPA, and the results are indicative of the immunostimulatory potential of the plant. It would be worthwhile to further evaluate *A. indica* on relevant immunomodulatory aspects, especially the *in vivo* studies in a poultry system.

## Authors’ Contributions

PR, TKA, and PG equally contributed in the conceptualization and design of the experiment. PR and TKA equally contributed in the experimentation, analysis and interpretation of the data and manuscript preparation. All the authors read and approved the final manuscript.
